# [Corrigendum] Saffron carotenoids inhibit STAT3 activation and promote apoptotic progression in IL‑6‑stimulated liver cancer cells

**DOI:** 10.3892/or.2024.8727

**Published:** 2024-03-27

**Authors:** Buyun Kim, Byoungduck Park

Oncol Rep 39: 1883–1891, 2018; DOI: 10.3892/or.2018.6232

Following the publication of the above article, an interested reader drew to the authors' attention that, in Fig. 1E on p. 1885, the STAT3 blots shown for the A549 and A2780 cell lines were strikingly similar, such that these data were possibly derived from the same original source where the panels were intended to show the results from differently performed experiments. Upon examining their original data, the authors have realized that an inadvertent error was made in assembling the data in the figure, and the STAT3 data shown correctly for the A549 cell line were erroneously copied across for the A2780 cell line.

The corrected version of Fig. 1, showing the correct STAT3 blot for the A2780 cell line in Fig. 1E, is shown on the next page. Note that this error did not affect the overall conclusions reported in the paper. All the authors agree with the publication of this corrigendum, and are grateful to the Editor of *Oncology Reports* for allowing them the opportunity to publish this. They also apologize to the readership for any inconvenience caused.

## Figures and Tables

**Figure 1.**
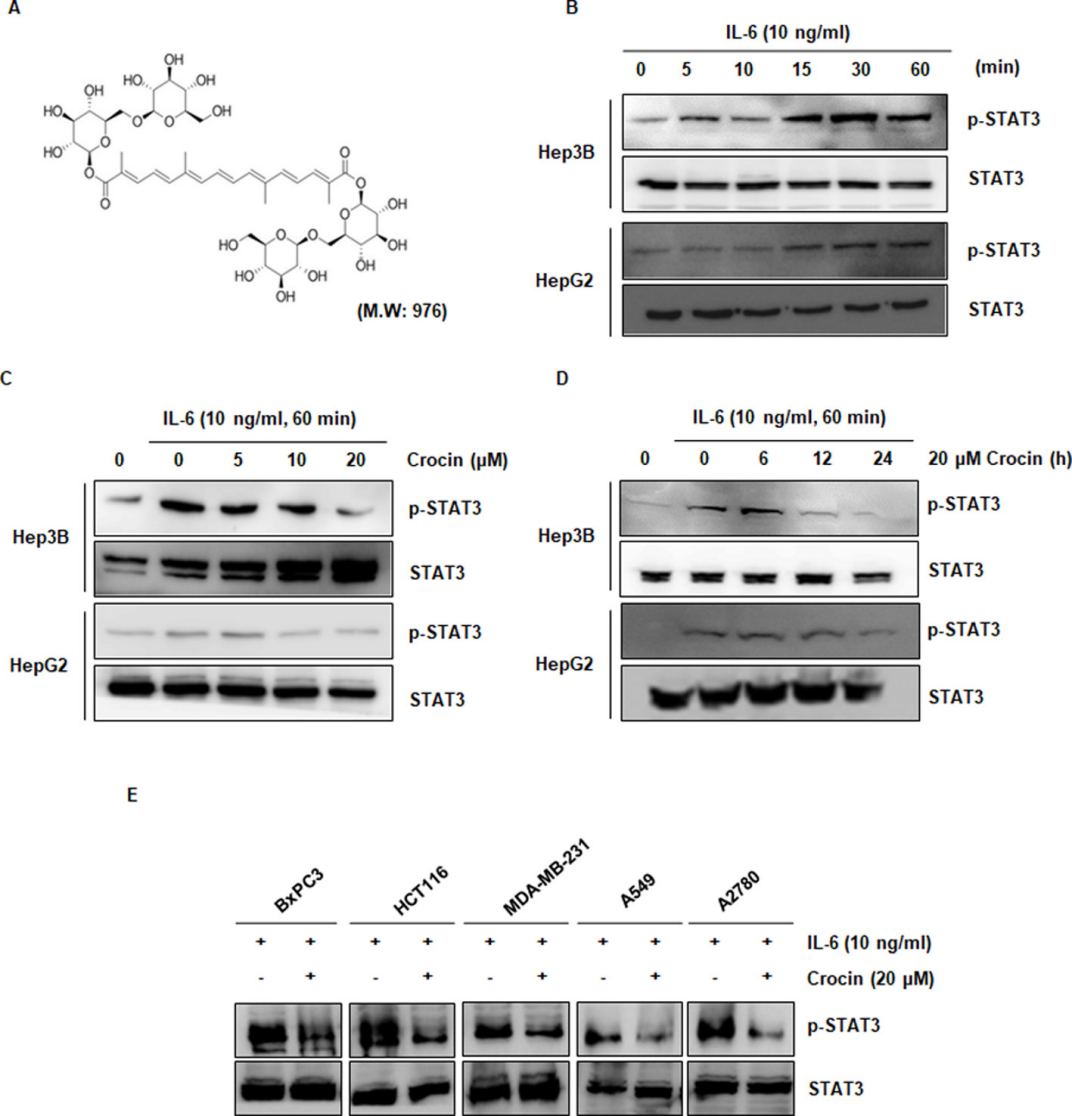
Crocin inhibits both inducible and constitutive STAT3 activation in liver cancer cells. (A) Chemical structure of crocin. (B) IL-6 induced STAT3 activity in liver cancer cells. Hep3B and HepG2 cells (1×10^6^/ml) were treated with IL-6 (10 ng/ml) for indicated time-points. Whole-cell extracts were prepared, and phosphorylated STAT3 was detected by western blotting as described in Materials and methods. (C) Crocin downregulated IL-6-induced phospho-STAT3 in a dose-dependent manner. Hep3B and HepG2 cells (1×10^6^/ml) were treated with the indicated concentrations of crocin for 24 h and then stimulated with IL-6 (10 ng/ml) for 60 min. Whole-cell extracts were prepared, and phospho-STAT3 was detected by western blotting. The same blots were stripped and reprobed with the STAT3 antibody to verify equal protein loading. (D) Crocin downregulated IL-6-induced phospho-STAT3 in a time-dependent manner. Hep3B and HepG2 cells (1×10^6^/ml) were treated with 20 µM crocin for the indicated time-points and then stimulated with IL-6 (10 ng/ml) for 60 min. Whole-cell extracts were prepared, and phospho-STAT3 was detected by western blotting. (E) Crocin suppresses phospho-STAT3 levels in breast and colon cancer cells. MDA-MB-231, HCT116, BxPC3, A549 and A2780 cells (1×10^6^/ml) were not or were treated with 20 µM of crocin and stimulated with IL-6 (10 ng/ml) for 24 h, after which whole-cell extracts were prepared, and phospho-STAT3 was detected by western blotting. The same blots were stripped and reprobed with the STAT3 antibody.

